# Trends in the Use of Guideline-Recommended Medications and In-Hospital Mortality of Patients with Acute Myocardial Infarction in a Chinese Population

**DOI:** 10.1371/journal.pone.0118777

**Published:** 2015-02-23

**Authors:** Jing Hu, Yanming Xie, Zheng Shu, Wei Yang, Siyan Zhan

**Affiliations:** 1 Evidence-based Medicine Center, School of Public Health, Peking University, Beijing, 100191, China; 2 Beijing Traditional Chinese Medicine Hospital, Capital Medical University, Beijing, Institute of Traditional Chinese Medicine, Beijing, 100010, China; 3 Institute of Basic Research in Clinical Medicine, China Academy of Chinese Medical Sciences, Beijing, 100700, China; 4 Center for Health Economics and Epidemiology Assessment, STATinMED (Beijing) International Healthcare Technology Assessment Co., LTD, Beijing, 100025, China; 5 Department of Epidemiology and Biostatistics, School of Public Health, Peking University Health Science Centre, Beijing, 100191, China; University of Minnesota, UNITED STATES

## Abstract

**Objective:**

Current practice guidelines recommend the routine use of several cardiac medications early in the course of acute myocardial infarction (AMI). Our objective was to analyze temporal trends in medication use and in-hospital mortality of AMI patients in a Chinese population.

**Methods:**

This is a retrospective observational study using electronic medical records from the hospital information system (HIS) of 14 Chinese hospitals. We identified 5599 patients with AMI between 2005 and 2011. Factors associated with medication use and in-hospital mortality were explored by using hierarchical logistic regression.

**Results:**

The use of several guideline-recommended medications all increased during the study period: statins (57.7%–90.1%), clopidogrel (61.8%–92.3%), β-Blockers (45.4%–65.1%), ACEI/ARB (46.7%–58.7%), aspirin (81.9%–92.9%), and the combinations thereof increased from 24.9% to 42.8% (P<0.001 for all). Multivariate analyses showed statistically significant increases in all these medications. The in-hospital mortality decreased from 15.9% to 5.7% from 2005 to 2011 (*P*<0.001). After multivariate adjustment, admission year was still a significant factor (OR = 0.87, 95% CI 0.79–0.96, *P* = 0.007), the use of aspirin (OR = 0.64, 95% CI 0.46–0.87), clopidogrel (OR = 0.44, 95% CI 0.31–0.61), ACEI/ARB (OR = 0.73, 95% CI 0.56–0.94) and statins (OR = 0.54, 95% CI 0.40–0.73) were associated with a decrease in in-hospital mortality. Patients with older age, cancer and renal insufficiency had higher in-hospital mortality, while they were generally less likely to receive all these medications.

**Conclusion:**

Use of guideline-recommended medications early in the course of AMI increased between 2005 and 2011 in a Chinese population. During this same time, there was a decrease in in-hospital mortality.

## Introduction

Despite a significant decrease in the incidence and mortality throughout the past decade, acute myocardial infarction (AMI) is still a major health problem worldwide, including in China [1,2]. *Report on Cardiovascular Disease in China*, *2010* [3] showed that the number of patients with MI was 2 million in 2010, and the expense for hospitalization in 2008 was CNY2.45 billion (about US$395 million) for AMI in China.

Numerous large-scale clinical trials have shown that the effectiveness of several cardiac medication treatments, such as dual antiplatelet therapy, β-Blockers, angiotensin-converting enzyme (ACE) inhibitors, or statins, substantially reducing mortality for patients with AMI [4–7]. These beneficial therapies have been widely incorporated into international and national guidelines including the Chinese guidelines [8–11]. While in China, some previous studies have documented the underuse of guideline-recommended medications for AMI patients [12–14]. However, the extent to which these medications use have changed over time and whether such changes are associated with improved clinical outcomes are still unknown. In our study, we chose several cardiac medications including aspirin, clopidogrel, β-Blockers, ACEI/ARB and statins, which were recommended for both acute ST-segment (STEMI) and non-ST-segment elevation myocardial infarction (NSTEMI) patients [10,11] in order to analyze the temporal trends, factors associated with early use of these medications and their impacts on in-hospital mortality of AMI patients in a Chinese population.

## Materials and Methods

### Data source

Data in this study are electronic medical records (EMR) from hospital information system (HIS) from 17 hospitals (including both Western Medicine (WM) and Traditional Chinese Medicine (TCM) hospitals) in China, which were standardized and integrated into a centralized data warehouse by Institute of Basic Research in Clinical Medicine, China Academy of Chinese Medical Science. All the 17 hospitals are level 3 institutions (major tertiary referral centers in provincial capitals and major cities) [15], which spread across 7 different provinces, including Beijing, Shenzhen, Guangdong, Shanxi, Fujian, Hebei and Jilin. There is a total of 2.42 million inpatients in the database, which was collected longitudinally from 2002 to 2011. It should be noted that in China, HIS were gradually implemented after 2005. In the 17 hospitals of our data warehouse, only one hospital has data between 2002 and 2004, so we did not include the data during this period for analysis. The majority of hospitals started to implement HIS in 2007 in our database, so the number of patients before 2007 were relatively smaller.

The data warehouse included baseline characteristics, diagnoses, treatment and lab examinations information of inpatients. Some text messages, such as previous MI, previous medication use prior to admission, and reperfusion therapies use, were not integrated into the database, so we could not analyze them.

### Ethics

The project received approval from Ethics Committee of Institute of Basic Research in Clinical Medicine, China Academy of Chinese Medical Science (2011 No. 11). Because data in our study are EMR from HIS, so we did not get written informed consent for included participants. To protect patient privacy, the patient records were anonymized prior to analysis.

### Study sample

The study population included all patients with AMI (n = 5,717), using International Classification of Diseases (ICD)-9 discharge diagnosis code of 410 and ICD-10 code of I21. Of those, we excluded hospitals with ≤30 AMI patients (number of hospitals = 3; n = 18), patients younger than 18 years old (n = 2), and patients admitted between 2002 and 2004 (number of hospitals = 1; n = 98). The final study population included 5,599 hospitalized patients from 14 hospitals—between January 2005 and May 2011, with a median of 294 patients per hospital.

### Data definitions

Drugs in the database were documented in product name, so we first transformed all of them to generic name, then classified to specific categories. Early medication use was defined as administration of drugs within the first 24 hours of hospitalization, which calculated as medication administration time minus admission time. The information of medical history was lacked in our database, so for the chronic diseases (hypertension, diabetes, hyperlipidemia, cancer, and renal insufficiency), we utilized co-morbidities information instead, the diagnosis of which were based on existing discharge diagnosis in the data warehouse. Age of patients was classified as <65, 65–74 and >75 years. Length of stay was calculated as the number of days from admission to discharge. The in-hospital mortality was defined as all-cause mortality during hospitalization.

### Statistical analysis

Patient characteristics, medication use, and in-hospital mortality were compared among years. Categorical variables were presented as frequencies and percentages, and Cochran-Armitage testing for trends was used to analyze time trends. For continuous variables, medians with Inter Quartile Range (IQR) were calculated, and simple linear regression was used to analyze time trends. It should be noted that we intended to analyze the trends for each year at first, however, the data volume before 2007 was relatively small. In the end, we combined the data between 2005 and 2007 to analyze the temporal trends for 2005 to 2007, 2008, 2009, 2010 and 2011 years.

We used hierarchical multivariate logistic regression (with hospital site as a random effect to account for clustering within hospitals) to analyze the factors associated with medication use. The hospital-leveled variable was type of hospitals (Western Medicine hospitals vs. Traditional Chinese Medicine hospitals), because some studies showed that the usage rate of β-Blockers and statins in TCM hospitals were lower than WM hospitals, while in-hospital mortality rate was higher than latter [14,16]. For the patient-leveled variables, admission year was incorporated first into the model, followed by patient characteristics (age, sex, insurance status) and co-morbidities.

To analyze the impact of treatment changes on in-hospital mortality, hierarchical logistic regression which included the random effects was also used; the independent variables in the model were type of hospitals, admission year, patient characteristics, co-morbidities, in-hospital complications (cardiogenic shock, heart failure, and Transient Ischemic Attack (TIA)/stroke) and medications within 24h of hospitalization (aspirin, clopidogrel, β-Blockers, statins, and ACEI or angiotensin receptor blockers [ARBs]). Odds ratios and 95% CIs were reported for each model.

A *P* value of less than 0.05 was considered statistically significant for all tests. All analyses were performed by using SAS software (SAS Institute, Cary, NC, USA).

## Results

### Patient characteristics

Over the observation period, the median age decreased from 68 to 64 years (*P*<0.001), the proportion of men increased from 72.7% to 77.2% (*P* = 0.0055). Patients with hypertension increased from 48.3% to 50.2%, but it was not statistically significant, the prevalence of diabetes mellitus and hyperlipidemia remained stable. The proportion of patients with heart failure increased from 23.7% to 49.1% (*P*<0.001). The length of stay decreased significantly (*P* = 0.0158) (Table 1).

**Table 1:** Patient characteristics of the study sample.

	Overall(n = 5599)	2005–2007(n = 1022)	2008–2009(n = 2541)	2010–2011(n = 2036)	*P* value for temporal trend
Demographics					
Age(Missing = 884), median y (25%-75%)	65 (55–74)	68 (56–76)	65 (55–74)	64 (54–73)	<0.001
Male sex (Missing = 230), %	4060(75.6%)	740(72.7%)	1922(75.7%)	1398(77.2%)	0.0055
Ethnic (Missing = 366), %					0.0163
Han	5155(98.5%)	990(97.7%)	2441(98.5%)	1724(99.0%)	
Others	78(1.5%)	23(2.3%)	37(1.5%)	18(1.0%)	
Occupation (Missing = 210), %					<0.001
Business/professional/clerical work	778(14.4%)	212(23.0%)	388(15.8%)	178(8.9%)	
Manual labourer	1039(19.3%)	48(5.2%)	363(14.8%)	628(31.2%)	
Others	3572(66.3%)	662(71.8%)	1436(69.4%)	1206(59.9%)	
Insurance status(Missing = 126), %					<0.001
Medical insurance	4103(75.0%)	612(61.6%)	1862(75.5%)	1629(80.8%)	
Self-pay/ no insurance	1349(24.7%)	376(37.8%)	590(24.0%)	383(19.1%)	
Others	21(0.4%)	6(0.6%)	13(0.5%)	2(0.1%)	
Co-morbidities, %					
Hypertension	2762(49.3%)	494(48.3%)	1245(49.0%)	1023(50.2%)	0.8471
Diabetes	1223(21.8%)	229(22.4%)	544(21.4%)	450(22.1%)	0.9209
Hyperlipidemia	944(16.9%)	143(14.0%)	495(19.5%)	306(15.0%)	0.7119
Renal insufficiency	318(5.7%)	71(7.0%)	130(5.1%)	117(5.7%)	0.1888
Cancer	143(2.6%)	45(4.4%)	72(2.8%)	26(1.3%)	<0.001
In-hospital Complications					
Cardiogenic shock	335(6.0%)	63(6.2%)	168(6.6%)	104(5.1%)	0.0651
Heart failure	2199(39.3%)	242(23.7%)	956(37.6%)	1001(49.1%)	<0.001
TIA/stroke	71(1.3%)	10(1.0%)	31(1.2%)	30(1.5%)	0.3265
Length of stay, median d (25%-75%)	12 (8–17)	14 (9–20)	12 (8–17)	11 (8–16)	0.0158

### Use of guideline-recommended medications

**Fig 1:**
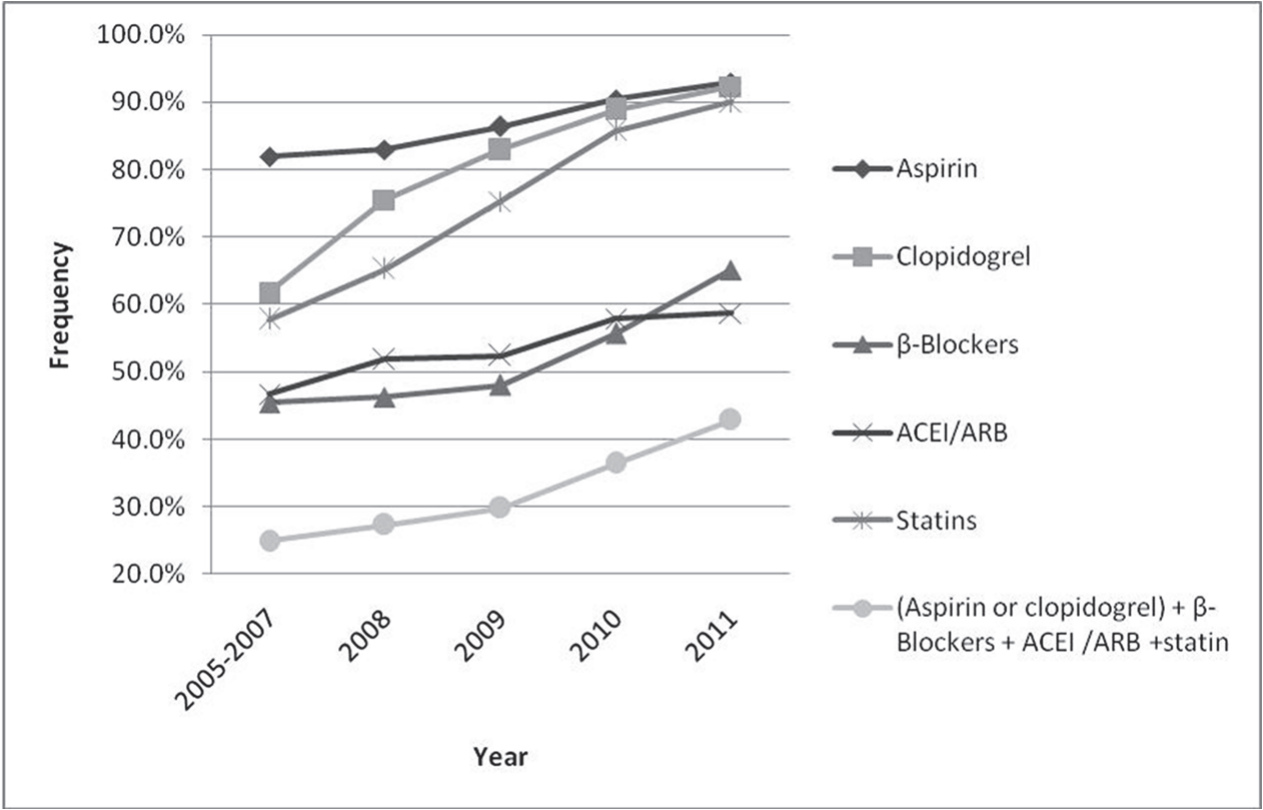
Unadjusted trends in the use of guideline recommended medications of patients with AMI.

The use of several medications within 24h of hospitalization all increased during the observation period (Fig. 1). The most notable improvement was seen in statins (from 57.7% to 90.1%), the next was clopidogrel (from 61.8% to 92.3%), others like aspirin increased from 81.9% to 92.9%, β-Blockers from 45.4% to 65.1%, and ACEI/ARB from 46.7% to 58.7% (*P*<0.001 for all). The proportion of patients who were prescribed 4-drug combination therapy (all 4 drugs of aspirin or clopidogrel, β-Blockers, ACEI/ARB, and statin) increased from 24.9% to 42.8% (*P*<0.001).

### Factors associated with medication use

Table 2 showed the results of hierarchical logistic regression that simultaneously controlled for patient characteristics and co-morbidities: temporal trends were statistical significant for all these guideline-based medications: aspirin (OR = 1.19, 95% CI 1.10–1.28), clopidogrel (OR = 1.62, 95% CI 1.47–1.78), β-Blockers (OR = 1.14, 95% CI 1.08–1.20), ACEI/ARB (OR = 1.13, 95% CI 1.07–1.19), statins (OR = 1.63, 95% CI 1.50–1.77), and 4-drug combination (OR = 1.18, 95% CI 1.11–1.25) (all *P*<0.001). Compared to patients younger than 65 years, patients with advanced age were significantly less likely to receive all these drugs. Men were more likely to receive aspirin, clopidogrel, β-Blockers and statins. Patients with hypertension were more likely to receive all these recommended medications, while patients with cancer and renal insufficiency were generally less likely to receive them. Patients with diabetes were less likely to receive clopidogrel. Patients with hyperlipidemia were more likely to receive aspirin, clopidogrel, β-Blockers, statins and 4-drug combination therapy.

**Table 2:** Multivariate analysis of factors associated with guideline-recommended medication use.

Model Covariate	Aspirin			Clopidogrel			β-Blockers			ACEI/ARB			Statins			4 drugs*		
	OR	95%CI	*P* value	OR	95%CI	*P* value	OR	95%CI	*P* value	OR	95%CI	*P* value	OR	95%CI	*P* value	OR	95%CI	*P* value
Admission year	1.19	1.10–1.28	<.0001	1.62	1.47–1.78	<.0001	1.14	1.08–1.20	<.0001	1.13	1.07–1.19	<.0001	1.63	1.50–1.77	<.0001	1.18	1.11–1.25	<.0001
Aged 65–74	0.67	0.54–0.84	0.0005	0.67	0.54–0.84	0.0005	0.72	0.62–0.84	<.0001	0.83	0.71–0.98	0.0247	0.66	0.55–0.81	<.0001	0.68	0.58–0.81	<.0001
Age > = 75	0.42	0.34–0.52	<.0001	0.37	0.29–0.48	<.0001	0.62	0.52–0.72	<.0001	0.70	0.59–0.83	<.0001	0.55	0.45–0.67	<.0001	0.53	0.43–0.64	<.0001
Male	1.45	1.19–1.75	0.0002	1.25	1.03–1.53	0.0262	1.17	1.02–1.35	0.0296	1.10	0.94–1.29	0.2154	1.35	1.13–1.62	0.001	1.11	0.94–1.30	0.2228
Medical insurance	1.20	0.98–1.46	0.0825	1.21	0.99–1.47	0.0595	0.92	0.80–1.05	0.2292	0.89	0.77–1.04	0.1434	0.85	0.71–1.01	0.0706	0.79	0.68–0.92	0.0028
Hypertension	1.50	1.25–1.79	<.0001	1.29	1.08–1.54	0.0043	1.46	1.29–1.65	<.0001	2.51	2.15–2.93	<.0001	1.37	1.17–1.60	<.0001	1.86	1.61–2.14	<.0001
Diabetes	0.99	0.81–1.22	0.9513	0.74	0.60–0.91	0.0039	1.04	0.91–1.20	0.5619	0.96	0.83–1.13	0.648	1.00	0.83–1.20	0.9801	1.09	0.93–1.28	0.2738
Hyperlipidemia	2.50	1.83–3.42	<.0001	3.19	2.33–4.37	<.0001	1.26	1.08–1.46	0.0034	1.10	0.93–1.30	0.2668	2.80	2.18–3.60	<.0001	1.35	1.15–1.60	0.0004
Cancer	0.11	0.08–1.16	<.0001	0.11	0.06–1.19	<.0001	0.30	0.19–0.46	<.0001	0.26	0.16–0.41	<.0001	0.15	0.09–0.24	<.0001	0.27	0.14–0.50	<.0001
Renal insufficiency	0.37	0.28–0.48	<.0001	0.35	0.25–1.49	<.0001	0.63	0.48–0.81	0.0003	0.64	0.49–0.85	0.0019	0.38	0.28–0.52	<.0001	0.47	0.33–0.66	<.0001

*: all 4 drugs of aspirin or clopidogrel, β-Blockers, ACE inhibitor/ARB, and statin.

### In-hospital mortality

**Fig 2:**
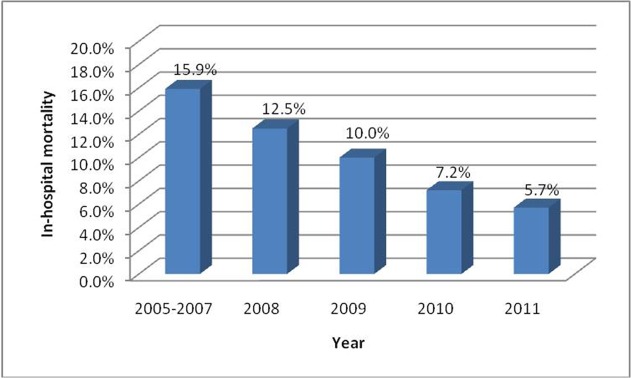
Unadjusted trends of in-hospital mortality rate of patients with AMI.

**Fig 3:**
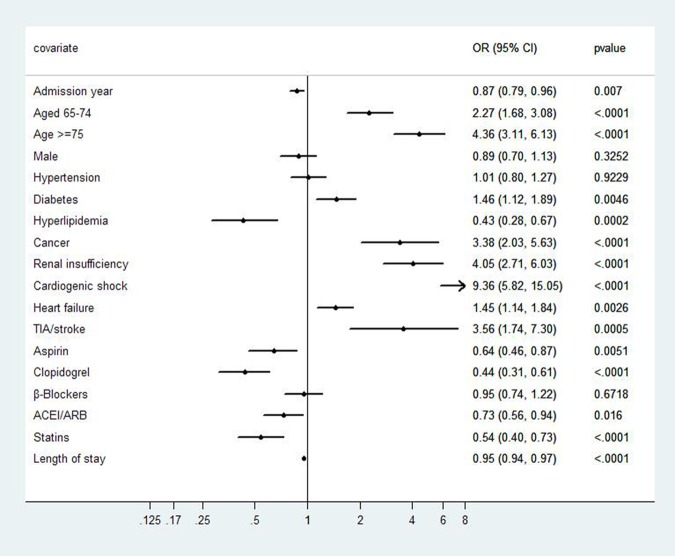
Multivariate analysis of factors associated with in-hospital mortality of patients with AMI.

Overall, there were 579 (10.3%) in-hospital deaths in the study population. Univariate analysis showed that the in-hospital mortality decreased from 15.9% to 5.7% during the observation period (*P*<0.001 for time trend) (Fig. 2). After multivariable adjustment, the admission year was still a significant factor (OR = 0.87, 95% CI 0.79–0.96, *P* = 0.007). Compared with patients aged younger than 65 years, patients aged between 65 and 74 years (OR = 2.27, 95% CI 1.68–3.08) and older than 75 years (OR = 4.36, 95% CI 3.11–6.13) had higher in-hospital mortality. Patients with diabetes (OR = 1.46, 95% CI 1.12–1.89), cancer (OR = 3.38, 95% CI 2.03–5.63) and renal insufficiency (OR = 4.05, 95% CI 2.71–6.03) had higher in-hospital mortality than their counterparts, while patients with hyperlipidemia (OR = 0.43, 95% CI 0.28–0.67) had lower mortality. Patients with in-hospital complications of cardiogenic shock, heart failure, and TIA/stroke all had higher in-hospital mortality. The use of aspirin (OR = 0.64, 95% CI 0.46–0.87), clopidogrel (OR = 0.44, 95% CI 0.31–0.61), ACEI/ARB (OR = 0.73, 95% CI 0.56–0.94) and statins (OR = 0.54, 95% CI 0.40–0.73) were associated with a decrease in in-hospital mortality (Fig. 3).

## Discussion

Our study demonstrated that there has been an increase over time in the early use of several guideline-recommended medications for AMI patients between 2005 and 2011 in a Chinese population. In China, the first AMI guideline was published in 2001 [17], after that, guideline for NSTEMI was updated in 2007 [10], and updated STEMI guideline was released in 2010 [11]. So maybe it was the potential reason for the increased uptake of recommended medications during the study period.

The increased trends of medication use for AMI patients in our study were consistent with some international studies [18,19]. The medication usage rates were generally lower than some prospective registry studies in China, such as the Clinical Pathways for Acute Coronary Syndromes in China (CPACS) study between 2004 and 2005 [12], and management of patients with STEMI in Liaoning province of China between 2009 and 2010 [13]. The use of aspirin, clopidogrel, β-Blockers and statin in these two studies were 98.1% and 96.6%, 63.0% and 81.9%, 74.7% and 66.0%, 88.5% and 90.1%, respectively. The main factor that might account for the low percentages was that we analyzed the medication use early in the course of AMI (within 24h of hospitalization) which based on guideline recommended, while both of these studies assessed the medical management during hospitalization.

The multivariate analysis in our study showed that the AMI mortality reduction was likely to be the consequence of the widespread use of guideline-recommended cardiac medications, with a caveat that the causality is difficult to ascertain. The finding was in accordance with other studies assessing in-hospital mortality for AMI patients over a similar period [20,21]. To improve quality of care and guideline adherence, some programs were developed throughout various countries, such as the ACC Guidelines Applied in Practice (GAP) program [22] and the AHA Get with the Guidelines-Coronary Artery Disease (GWTG-CAD) program [23]. In recent years, China has been gradually making efforts in developing some programs, like Bridging the Gap on CHD Secondary Prevention in China project (BRIG) in 2006 [24], and CHINA Patient-Centered Evaluative Assessment of Cardiac Events: Prospective Study of Acute Myocardial Infarction (PEACE-P-AMI) [25], which initiated in 2012. Findings of these studies will accelerate evidence-based clinical practice and policy making, and improve patients’ outcomes in China.

Our study found that those AMI patients with older age, renal insufficiency and cancer have higher mortality, so their absolute benefit from treatment is more prominent than their counterparts. However, multivariate analysis showed that these patients were less likely to receive these guideline-based medications. Previous observations [26,27] have revealed that higher-risk patients are less likely to receive guideline recommended therapies, which has been termed the “risk-treatment paradox”. This paradox has been attributed to the following reasons: physicians may be uncertain about the risk/benefit ratio in patients at higher risk who are generally under-represented in clinical trials; elderly patients are more likely to have a variety of additional co-morbidities and a greater intolerance to medications, so physicians may be concerned about adverse events with treatment. In clinical practice, clinicians should be encouraged to use some objective data to weigh risks and benefits carefully before withholding evidence-based therapies in these patients.

Patients with hyperlipidemia had lower in-hospital mortality in our study, which was also found in Gierlotka et al’s study [21]. Some studies [28,29] had shown the cholesterol paradox (higher low density lipoprotein levels were related to better clinical outcomes) in AMI patients. Wang [29] indicated that the paradox might be related to confounding, where this protective association persisted after multifactor adjustment, so it was attributed to some unmeasured confounders that may not be captured by observational databases. In addition, AMI patients with hyperlipidemia are younger (which was verified in our database) [29], and hyperlipidemia may reflect better nutritional and health status, which are more likely to associate with better tolerance of acute medical stress.

### Strengths and limitations

The data of this study were derived from a centralized data warehouse integrated from HIS of 14 Chinese hospitals. To the best of our knowledge, this is the first study evaluating the implementation of guidelines in the ‘‘real world’’ practice of patients with AMI using HIS in China. Compared to prospective registry studies, using data located in the HIS is a cost-effective and time-saving method to measure the quality-of-care indicators, because these data are more readily accessible.

However, as a retrospective observational study, we could only utilize the existed information in the data warehouse; the major limitation is that some important treatment measures during hospitalization (e.g., method of revascularization/Door-to-Balloon time) and some major contraindications to medication use (e.g., allergy to medications) were lacked, so we were unable to comment on them. In addition, although our study showed that early use of recommended medicines was associated with a decrease in in-hospital mortality, the improved mortality may be related to a number of factors that were not included in our data warehouse, for example, pre-admission use of cardiovascular medicines, STEMI vs NSTEMI diagnosis, and changes in trends in use of reperfusion therapies. Therefore, even after multivariable adjustment, the results could be biased by the lack of potentially important parameters. Consequently, the findings of our study should be interpreted with caution.

## Conclusions

Our analysis demonstrated that use of guideline-recommended cardiac medications early in the course of AMI has generally increased between 2005 and 2011 in a Chinese population. More importantly, these treatment changes were associated with a decrease in in-hospital mortality. Patients with older age, renal insufficiency and cancer have higher mortality than their counterparts, while these patients were less likely to receive all the medications.
